# How to cushion economic recession caused by the COVID-19 pandemic: Fiscal or monetary policies?

**DOI:** 10.3389/fpubh.2022.960655

**Published:** 2022-10-28

**Authors:** Ya Wu, Yu Luo

**Affiliations:** School of Economics, Jinan University, Guangzhou, China

**Keywords:** COVID-19, economic recovery, fiscal policy, monetary policy, computable general equilibrium model

## Abstract

The outbreak of the COVID-19 pandemic has brought the global economy to a crisis: how to choose the optimal policy tools to cope with the external impacts has attracted worldwide attention. The research evaluates the effects of China's fiscal and monetary policies in promoting economic recovery by establishing a CGE model. Five representative countermeasures such as exempting value-added tax (VAT) and cutting loan rates are studied. The results indicate that: from the aspect of fiscal policies, increasing investment shows a better effect in boosting economy compared with exempting VAT and increasing medical care expenditures; however, the policy also causes price inflation (+0.45%) and crowding-out of enterprise investment (−0.03%). From the aspect of monetary policies, providing targeted loans to enterprises has a better boosting effect on economy compared with cutting loan rates. In the choice between fiscal or monetary policies, fiscal policies exert better effects (household income, +0.95%) when taking the improvement of residents' welfare as the objective. If taking promoting recovery of enterprises and boosting the economy as objectives, monetary policies are found to be better (GDP, +1.99%). Therefore, fiscal and monetary policies should be guided by different objectives and allowed to work in a synergistic manner.

## Introduction

The unexpected outbreak of the COVID-19 in 2020 has wreaked havoc across the world. The “Black Swan” event has dealt a huge blow to the global economy. The World Economic Outlook released by the International Monetary Fund (IMF) in January 2021 indicates that global economy had been recessed by 3.5% in 2020.^1^ This has become the most severe crisis since the Great Depression. To promote rapid recovery of global economy from the catastrophe, countries around the world need to use policy interventions to cope with the public emergency while containing the pandemic.

The Chinese Government has introduced a series of policies to promote economic recovery following the outbreak of the COVID-19 pandemic. From the fiscal aspect, the deficit-to-GDP ratio of China in 2020 exceeded 3%, which increased by one trillion yuan from its 2019 level. At the same time, one trillion yuan of government bonds for COVID-19 control and 3.75 trillion yuan of special local government bonds were also issued. These funds focus to cutting taxes for enterprises and expanding investment in public health and infrastructure.^2^ From the perspective of monetary policies, the People's Bank of China has cut the reserve requirement ratio three times and the loan prime rate two times and earmarked 1.8 trillion yuan for re-lending and rediscount in 2020.^3^ These policies have provided enough monetary support for the complete resumption of production.

With the mitigation of the pandemic and implementation of various policies, China's economic growth has turned from negative to positive since the third quarter of 2020, showing a “V-shaped” recovery trend. The IMF and the Organization for Economic Cooperation and Development both identify China as the only major economy with positive economic growth in 2020. While containing the pandemic, how much do fiscal and monetary policies play their roles in promoting economic recovery? What is the optimal fiscal or monetary policy for coping with this public crisis? The economic impact analysis and economic policy interventions for tackling public crises are important for the academe and governmental departments. Therefore, in the present research, we summarize key fiscal and monetary measures taken in China since the outbreak of the pandemic. By adopting the computable general equilibrium (CGE) model, the research quantitatively evaluates implementation effects of fiscal and monetary policies.

The existing studies are usually based on the traditional CGE model to examine the impact of the COVID-19 outbreak on the macro-economy (1–5), there are also a few studies that consider fiscal policies at the same time, such as exempting value-added tax (4, 6) or expanding government investment (7, 8) on economic recovery. However, in the face of the economic recession caused by the pandemic, monetary stimulus and fiscal stimulus are often carried out simultaneously, so it is necessary to make a comparative study of their effects. The contribution of the paper is as follows: the traditional CGE model usually includes five modules: resident, enterprise, government, commodity production and market equilibrium (9). The research based on the traditional CGE model can investigate the impact of price fluctuation (10, 11), disaster impact (12, 13) and finance policy (14) on the social economy. However, since the traditional CGE model does not include the lending behavior of the financial market, the implementation effect of monetary policies such as interest rate and credit cannot be investigated. Therefore, based on the five modules of the traditional CGE model, this study adds a financial module, which complements the lending behavior of banks to enterprises in the market. This setup is convenient to examine the effects of monetary policy tools such as reducing loan interest rates and expanding credit scale. Based on the above model innovation, this paper can compare the implementation effects of fiscal policy and monetary policy, which effectively makes up for the shortcomings of existing research, and better inspires policy making under public health risk.

## Literature review

In recent years, the impacts of major public health emergencies (PHE) on the macro-economy and their policy responses have gradually attracted attention among scholars. The impacts of PHE on the macro-economy are generally measured using the CGE model. The single-country CGE model (1, 4, 5, 12, 15), global trade analysis project model (16, 17), and multi-country, or multi-sector intertemporal general equilibrium model (18) can be used according to differences in transmission ranges of pandemic diseases and research demands. Some studies place emphases on discussing the impacts of PHE on the economy from the demand side, which are mainly relevant to highly contagious diseases such as severe acute respiratory syndrome infections (18). Some studies focus on the impacts of PHE on the economy from the supply side, which are mainly related to highly fatal diseases such as Ebola infections (19). There are also studies paying attention to the effects of factors in both the supply and demand sides on the economy (12).

Although existing research has explored impacts of PHE on macro-economy, the research into how economic policies, particularly fiscal and monetary policies, promote economic recovery remains scarce. For the effects of fiscal policies, some scholars have studied effects of fiscal policies on household consumption and enterprise investment (20–24). Some scholars have studied the effect of fiscal policies on prices (25, 26). As for the effects of monetary policies, many scholars have compared the effect of quantitative monetary policies and price monetary policies (27–31). For the choice between fiscal and monetary policies, some scholars proposed that compared with a single policy, a combination of fiscal and monetary policies can more substantially mitigate macro-economic fluctuations (21). Monetary policies should aim to stabilize prices while fiscal policies should aim to stabilize output (32). The price-oriented monetary policy and expenditure-oriented fiscal policy are the optimal combination from the perspective of social welfare (29).

Fewer studies have comparatively analyzed the impact of fiscal and monetary policies on economic recovery in the face of a catastrophe. Chen et al. (33) constructed an RBC model involving catastrophic factors and found that increasing fiscal subsidies can alleviate catastrophic impacts on the economy. Zhao et al. (34) found that government productive expenditures can cushion the impacts of a disaster on consumption and output by using a dynamic stochastic general equilibrium (DSGE) model. Chao (35) used the DSGE model to study the transmission mechanism of fiscal and monetary policies during a catastrophe, finding that monetary policies can significantly shorten the recovery time of output, consumption, and investment under the impact. In addition, some scholars investigated the impact of fiscal policies on economic recovery and environmental pollution. For example, Lahcen et al. (7) used the CGE model to discuss the green recovery path of the economy under the impact of the COVID-19 pandemic. The research shows that expanding investment in environmental protection can effectively promote economic recovery and reduce CO_2_ emissions. Xu and Wei (36) constructed a dynamic CGE model and proposed that the policy of large-scale tax reduction and fee reduction can alleviate the impact of the COVID-19 pandemic on the macro-economy. Unfortunately, this policy will also promote fossil energy consumption, greenhouse gas and pollutant emissions.

Compared with existing studies, this paper supplements and extends the following aspects thereof: the first is method selection. The research uses CGE model for analysis. Compared with the DSGE model, CGE model has advantages such that it can be used to examine the correlation and dependence of various sectors and micro-economic agents. The second is policy choice, which closely follows the current situation. This paper reviews China's fiscal and monetary measures since the outbreak and quantifies differences in their effectiveness and mechanisms using CGE models. The third is the assessment of policy effects, for which multiple perspectives are selected. The research demonstrates the possible positive and adverse effects of fiscal and monetary policies from perspectives of the society, residents, and enterprises, providing a multi-view theoretical basis for policy formulation in the face of future catastrophic risks.

## Methodology

### Equations of the CGE model

The CGE model has become the mainstream model for studying the effects of macro-policies on an economy (37, 38). Based on China's Input-Output Table, the research establishes a consistent standard data set for the CGE model by referring to the method of compilation of Fan and Zheng (39) and Zhao and Wang (40) for the social accounting matrix (SAM). The model comprises five micro-economic agents (resident, enterprise, government, bank, and foreign department), two production factors (labor and capital), and six modules (production, resident, enterprise, government, finance, and market equilibrium modules).

In setting the equations in the CGE model we refer to Fan et al. (13), and a finance module is added to the traditional CGE model. Compared with the traditional CGE model, the setting enables one to study effects of loans and lending in the financial market on the overall equilibrium. The model is solved by the GAMS and equations for each module are set as follows:

#### Production module

The equation setting of the production module in this paper is consistent with the traditional CGE model. Readers can refer to Lofgren et al. (9) for a detailed description of model specification in production and trade block section.

#### Resident module

The total income of resident has four sources: labor income, capital income, deposit interest income and transfer payments from enterprise, government and foreign departments. The total expenditure of resident is reflected in three aspects: consumption, income tax and savings.


(1) $$\begin{array}{l} YH=WL\cdot sf_{hl}\cdot QLS+WK\cdot sf_{hk}\cdot QKS+HST\\ \;+tr_{hent}+tr_{hgov}+tr_{hrow}\cdot EXR \end{array}$$

(2) $$\begin{array}{l} \;EH\;=\;ti_{h}\;\cdot \;YH\;+\;PQ_{c}\cdot \;QH_{c}\;+\;HSAV \end{array}$$

*YH*, *EH*, *HST* and *HSAV* respectively represent resident's total income, total expenditure, deposit interest income and resident savings; *PQ* and *QH* represent the price of commodities and the demand for commodities; *QLS* and *QKS*, respectively, represent the total supply of labor and capital; *EXR* represents the exchange rate; *sf* represents distribution coefficient; *tr* represents transfer payment; *ti* represents tax rate.

#### Enterprise module

The total income of enterprise comes from four parts: capital income, enterprise loan, deposit interest income and transfer payments from government and foreign departments. The total expenditure of enterprise includes enterprise income tax, transfer payment to resident department, fixed asset investment, savings and loan interest expense.


(3) $$\begin{array}{l} YE=sf_{entr}\cdot \;WK\cdot QKS+LNE+EST+tr_{egov}\\ \;+tr_{erow}\cdot EXR \end{array}$$

(4) $$\begin{array}{l} \;EE\;=\;{\;ti}_{ent}\;\cdot \;YE\;+\;tr_{hent}\;+\;EIV\;+\\ ESAV\;+\;ELT \end{array}$$

*YE*, *EE*, *EIV*, and *ESAV* are, respectively, enterprise income, expenditure, fixed asset investment and enterprise savings; *LNE*, *ELT*, and *EST*, respectively, represent enterprise loan, loan interest expense and deposit interest income.

#### Government module

The total revenue of the government comes from various taxes, deposit interest and transfer payment from foreign department. Government revenue is mainly used for government purchase expenditure, investment, savings and transfer payment to enterprise and resident.


(5) $$\begin{array}{l} YG=\underset{a}{\sum^{\text{​}}}(tl_{a}\cdot WL\cdot QLD_{a}+tk_{a}\cdot WK\cdot QKD_{a})+ti_{h}\cdot YH\\ \;+ti_{ent}\cdot YE+\underset{a}{\sum^{\text{​}}}ta_{a}\cdot PA_{a}\cdot QA_{a}\\ \;+\underset{c}{\sum^{\text{​}}}tm_{c}\cdot \;pm_{c}\cdot QM_{c}\cdot \;EXR+GST+tr_{grow}\cdot \;EXR \end{array}$$

(6) $$\begin{array}{lll} EG & = & \underset{c}{\sum }PQ_{c}\cdot {QG}_{c}+tr_{hgov}+tr_{egov}+GIV+GSAV \end{array}$$

*YG*, *EG*, *QG* and *GIV* represent government income, expenditure, government purchase and fixed asset investment, respectively; *GSAV* and *GST* represent government savings and deposit interest income. *PA* and *QA* represent the price and quantity of production activities; *QM* represents the quantity of imported commodities; *tl* *and* *tk* represent the labor and capital value-added rate*;* *ta* and *tm* represent the production tax rate and import tax rate; *pm* represents the international price of export commodities.

#### Finance module

Interest on deposits and loans of various departments:


(7) $$\begin{array}{l} HST\;=\;HSAV\cdot {\;is}_{h} \end{array}$$

(8) $$\begin{array}{l} EST\;=\;ESAV\;\cdot \;is_{e} \end{array}$$

(9) $$\begin{array}{l} GST\;=\;GSAV\;\cdot \;is_{g} \end{array}$$

(10) $$\begin{array}{l} FST\;=\;FSAV\;\cdot {\;is}_{f}\;\cdot \;EXR \end{array}$$

(11) $$\begin{array}{l} ELT\;=\;LNE\;\cdot {\;il}_{e} \end{array}$$

The income of the bank is the total amount of deposits and loan interest paid by each department, while the expenditure of the bank is the total amount of loans and deposit interest paid by each department in the bank:


(12) $$\begin{array}{l} YB\;=\;HSAV\;+\;ESAV\;+\;GSAV\;+\;FSAV\;\cdot \;EXR\;+\;ELT \end{array}$$

(13) $$\begin{array}{l} EB\;=\;LNE\;+\;HST\;+\;EST\;+\;GST\;+\;FST\;+\;BIV \end{array}$$

*YB* and *EB*, respectively, represent income and expenditure of the bank; *BIV* represents bank loan investment; *FSAV* and *FST* represent foreign department savings and deposit interest income; *is* and *il*, respectively, represent deposit and loan interest rates.

#### Market equilibrium module

When the market reaches general equilibrium, the supplies of commodity market and factor market are required to be equal to the demands, and the expenditures of micro-departments of resident, enterprise and bank are required to be equal to the incomes. In addition, the model satisfies the assumption of a small open economy, where the domestic economy is a small part of the world. The domestic market price does not affect the international market price and imported goods can only accept the international market price; Trade balance (capital flow) is exogenous, capital flows in and out freely at a fixed world interest rate, and the real exchange rate changes make the international balance of payments.

### Data of the CGE model

To establish the data set of the CGE model, that is, China's SAM, the Input-Output table in 2017 (the latest version published in China), flow-of-funds table in 2017, Finance Yearbook of China in 2018, and international balance of payments in 2017 are used. Therein, the intermediate input and import come from the Input-Output table in 2017; labor reward, capital gain, net product tax, labor income, direct tax, resident savings, enterprise savings, government consumption, gross investment and enterprise loan are derived from the flow-of-funds table in 2017; corporate tax and government transfer payments to resident and enterprise are derived from the Finance Year Book of China in 2018; export, net labor reward abroad, and net foreign transfer payments to resident come from the international balance of payments data in 2017. Appendix A shows the detailed sources and initial values of all data in the SAM. Appendix B provides the balanced macro-SAM.

Besides, some important parameters are also involved in the CGE model, including the substitution, expenditure, and prices elasticities, whose coefficients are set by referring to the GTAP data base version 10 (41). To be specific, the coefficients of capital-labor substitution elasticity, substitution elasticity between domestic products and imports, price elasticity of export demands, and expenditure elasticity of resident for different goods are separately set to 0.9, 3.0, 4.0, and 1.5.

## Empirical analysis

### Overviews of fiscal and monetary policies during the COVID-19 pandemic

The research summarizes relevant fiscal and monetary policies implemented in China since the outbreak of the pandemic. Among the policies introduced, fiscal policies are mainly used to cope with the pandemic impact by means of reducing taxes and fees, expanding government investment, and increasing medical care expenditure (Table 1).

**Table 1:** Overviews of fiscal policies during the COVID-19 pandemic.

**Policy tools**	**Content**
Tax reduction	Exempt VAT on services such as public transportation, restaurants and hotels, tourism and entertainment, and culture and sports. Reduce VAT for small-scale taxpayers.
Fee reduction	Exempt small and medium-sized enterprises from payment of pension, unemployment and industrial injury insurance units. Reduce industrial and commercial electricity prices by 5%.
Expand investment	Increase investment in new infrastructure construction. Develop a new generation of information networks, expand 5G applications, and promote new energy vehicles. Strengthen transportation, water conservancy and other major projects. Increase the national railway construction capital.
Increase medical and health expenditure	Increase investment in research and development of vaccines, medicines and rapid detection technologies. Financial support is implemented for the personal expenses of confirmed patients, with a 60% subsidy from the central government.

Source: From the Report on the Work of the Government in 2020 and the Ministry of Finance of the People's Republic of China.

In terms of monetary policies, apart from conventional approaches (cutting the reserve requirement ratio, cutting interest rates, and opening market) in response to financial crises, unconventional policies are also developed to cope with the pandemic, such as introducing re-lending and rediscount for pandemic prevention and control (Table 2).

**Table 2:** Overviews of monetary policies during the COVID-19 pandemic.

**Policy tools**	**Content**
Opening market	The short-term reverse repurchase in the opening market was carried out from February 3rd to 4th, 2020, and the winning bid rate of 7-day reverse repurchase operation decreased by 30 basis points compared with the previous period.
Standing leading facility	The standing leading facility interest rate was lowered by 30 basis points on April 10th, 2020.
Medium-term loan facility	The one-year medium-term lending facility was carried out on February 17th and April 15th, 2020, and the winning bid rate dropped by 10 basis points and 20 basis points, respectively, compared with the previous period.
Reserve requirement ratio	The reserve requirement ratio of financial institutions was lowered by 0.5 percentage points on January 6th, 2020, releasing about 800 billion yuan of long-term funds. The targeted cuts to reserve requirement ratio of inclusive finance was implemented, giving 0.5 or 1.5 percentage points of preferential reserve requirement ratio to qualified institutions on March 16th, 2020, and releasing about 550 billion yuan of long-term funds.
Re-lending and rediscount	A special re-lending of 300 billion yuan for pandemic prevention was set up on January 31st, 2020. The 500 billion yuan and 1 trillion-yuan re-lending and rediscount was issued on February 26th and April 20th, respectively.
Loan prime rate	The one-year loan prime rate was lowered by 10 basis points and 20 basis points, respectively, on February 20th and April 20th, 2020, and the five-year loan prime rate was lowered by 5 basis points and 10 basis points, respectively.

Source: From the Report on China's Monetary Policy Implementation in the Fourth Quarter of 2020 and the People's Bank of China.

### The impact of fiscal and monetary policies on China's macro-economy

To analyze quantitatively the effects of macro-policies in economic recovery, three fiscal policies, including exempting VAT for designated sectors, expanding government investment, and increasing medical care expenditure, as well as two monetary policies, including cutting loan rates and increasing the scale of lending are chosen for simulation. After introducing each policy into the model, the simulation results of corresponding policy can be obtained by adjusting the VAT rate, government investment in fixed assets, government purchases, interest rate on enterprise loan, and scale of enterprise loan in the model. The parameter settings of the model are as follows:

Fiscal policy 1: Exempting VAT for designated sectors. The VAT rate on sectors including public transportation, restaurants and hotels, tourism and entertainment, and culture and sports is reduced to 0%.^4^

Fiscal policy 2: Expanding government investment. Government investment in traditional infrastructure construction (transportation and water conservation) is increased to 20.7%,^5^ and that in new infrastructure construction represented by communication and information technologies is increased to 34.5%.^6^

Fiscal policy 3: Increasing medical care expenditure. Government expenditure on the health care sector is increased by 6.96%.^7^

Monetary policy 1: Cutting loan rates. The interest rate on corporate loans is reduced from 5.12% to 4.61%.^8^

Monetary policy 2: Increasing loan scale. The scale of corporate lending is expanded to 12.6%.^9^

**Figure 1:**
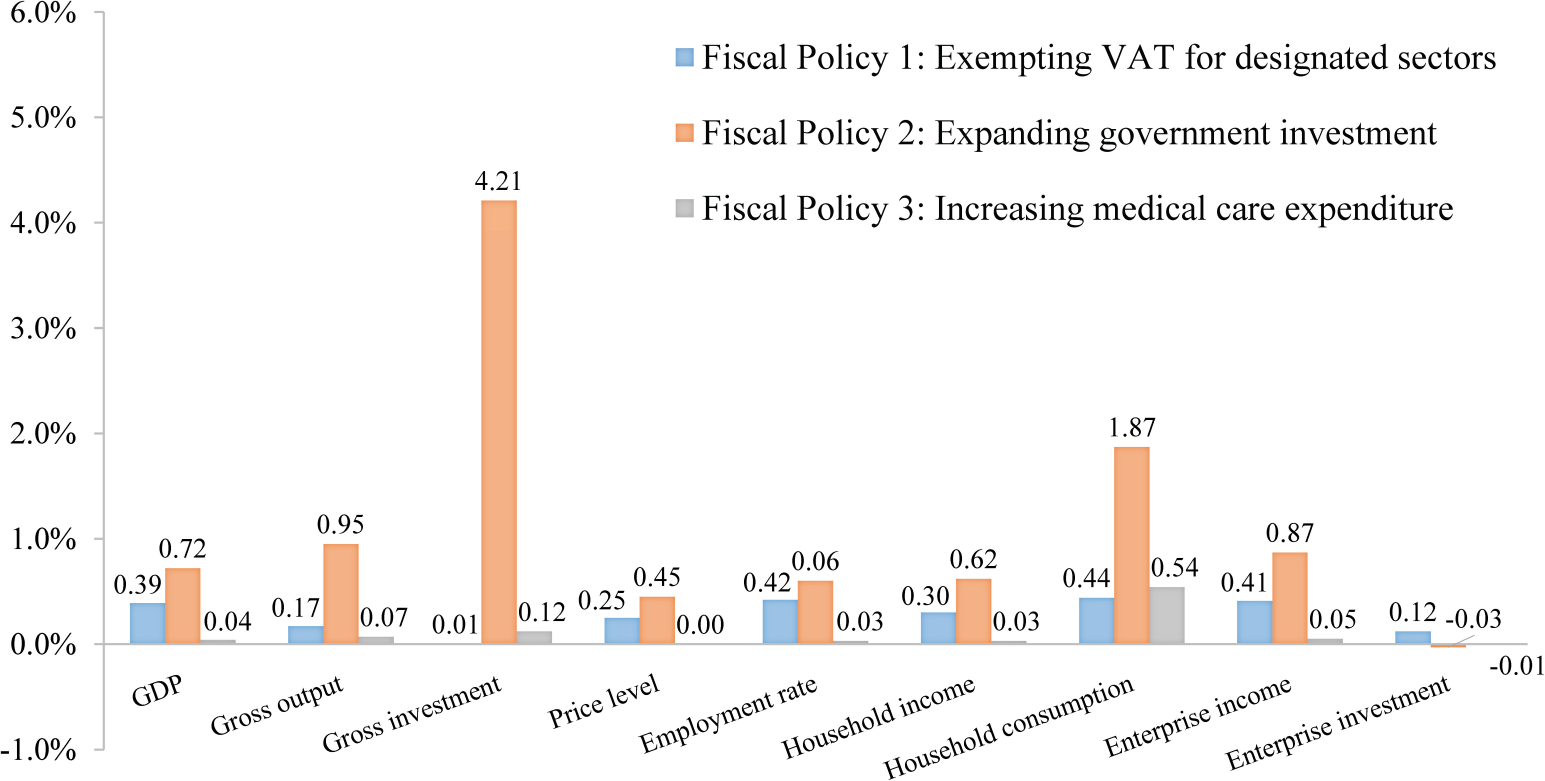
The effects of fiscal policies on macro-economy (Unit: %).

Figure 1 shows the actual effects of various fiscal policies. Expanding government investment has a significant effect in boosting the economy (GDP, +0.72%). On the one hand, it improves aggregate societal demand, making gross investment and consumption separately increase by 4.21% and 1.87%. On the other hand, the policy also enhances social aggregate supply, making the gross output increase by 0.95%. While it is worth noting that the government investment exerts much larger effects on the demand side than the supply side in the short term, so the policy also significantly increases prices. From the perspective of residents, expanding government investment can ease the impact of the pandemic on household income and consumption. Attributed to government investment, the employment rate, household income, and consumption separately increase by 0.60%, 0.62%, and 1.87%. From the aspect of enterprises, expanding government investment increases revenue (+0.87%), while crowding-out investment (−0.03%). This is because the growth of revenue stimulates trade demand, which puts upward pressure on interest rates. As a result, enterprises will invest less.

Exempting VAT for designated sectors exerts a weaker effect in boosting the economy (GDP, +0.39%) compared with expanding government investment, while it alleviates some impacts of the pandemic on employment (+0.42%) and consumption (+0.44%). Public transportation, restaurants and hotels, and tourism and entertainment are the sectors most heavily affected by the pandemic (these stagnated during lockdown). Exempting VAT for these sectors directly reduces their operating cost, avoids large scale bankruptcy and unemployment, and promotes lowering of price of goods and services provided by these sectors, which to some extent stimulates consumption.

Increasing medical care expenditure has the weakest effect in boosting economy (GDP, +0.04%). The core objective of the government when increasing medical care expenditure is to rescue patients, curb the spread of COVID-19, and ensure public welfare. The effects of increasing medical care expenditure on the price, employment, and income are nearly 0%; it only has a significant effect in boosting consumption (+0.54%). This is because increasing medical care expenditure to provide a series of free medical services (free testing, free treatment, and free vaccination against COVID-19) nationwide relieves people's concern about future uncertainties. Therefore, residents reduce precautionary savings correspondingly while increasing their current levels of consumption.

**Figure 2:**
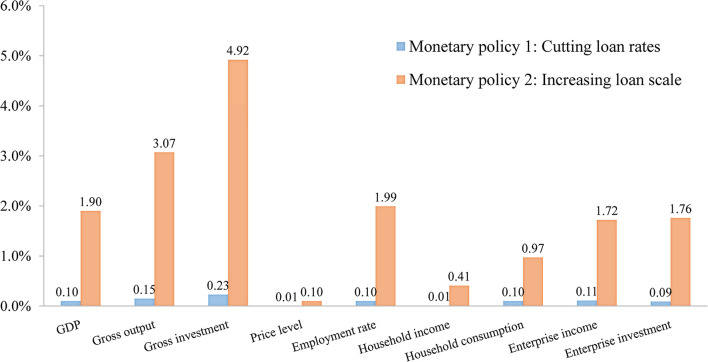
The effects of monetary policies on macro-economy (Unit: %).

Figure 2 shows the actual effects of various monetary policies: providing targeted loans to enterprises directly (Increasing loan scale) shows a better effect in boosting the economy compared with cutting loan rates across the board. Cutting loan rates across the board only enables 0.10% growth in GDP, while the other policy stimulates GDP growth of 1.90%. On the demand side, increasing the scale of lending improves gross investment and consumption by 4.92% and 0.97% separately; on the supply side, increasing the scale of lending increases gross output by 3.07%. This indicates that the policy can promote economic recovery simultaneously from both the supply and demand sides. From the perspective of enterprises, increasing the scale of lending stimulates enterprise investment, which enables enterprise investment and revenue to increase separately by 1.76% and 1.72%. This shows that increasing the scale of lending not only can provide loan funds to enterprises to help them overcome difficulties in the short term, but also stimulates enterprise investment, thus promoting their development in the long term.

**Figure 3:**
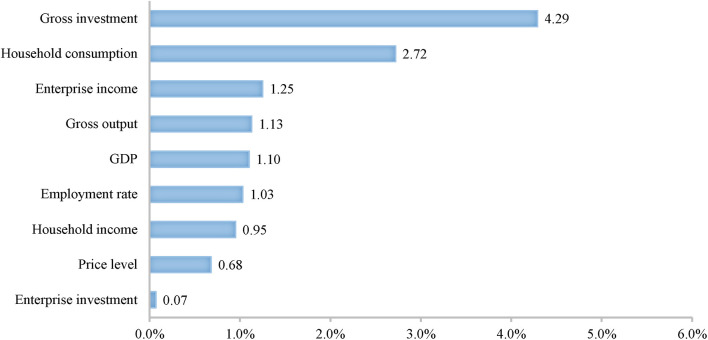
The total effects of fiscal policies on macro-economy (Unit: %).

**Figure 4:**
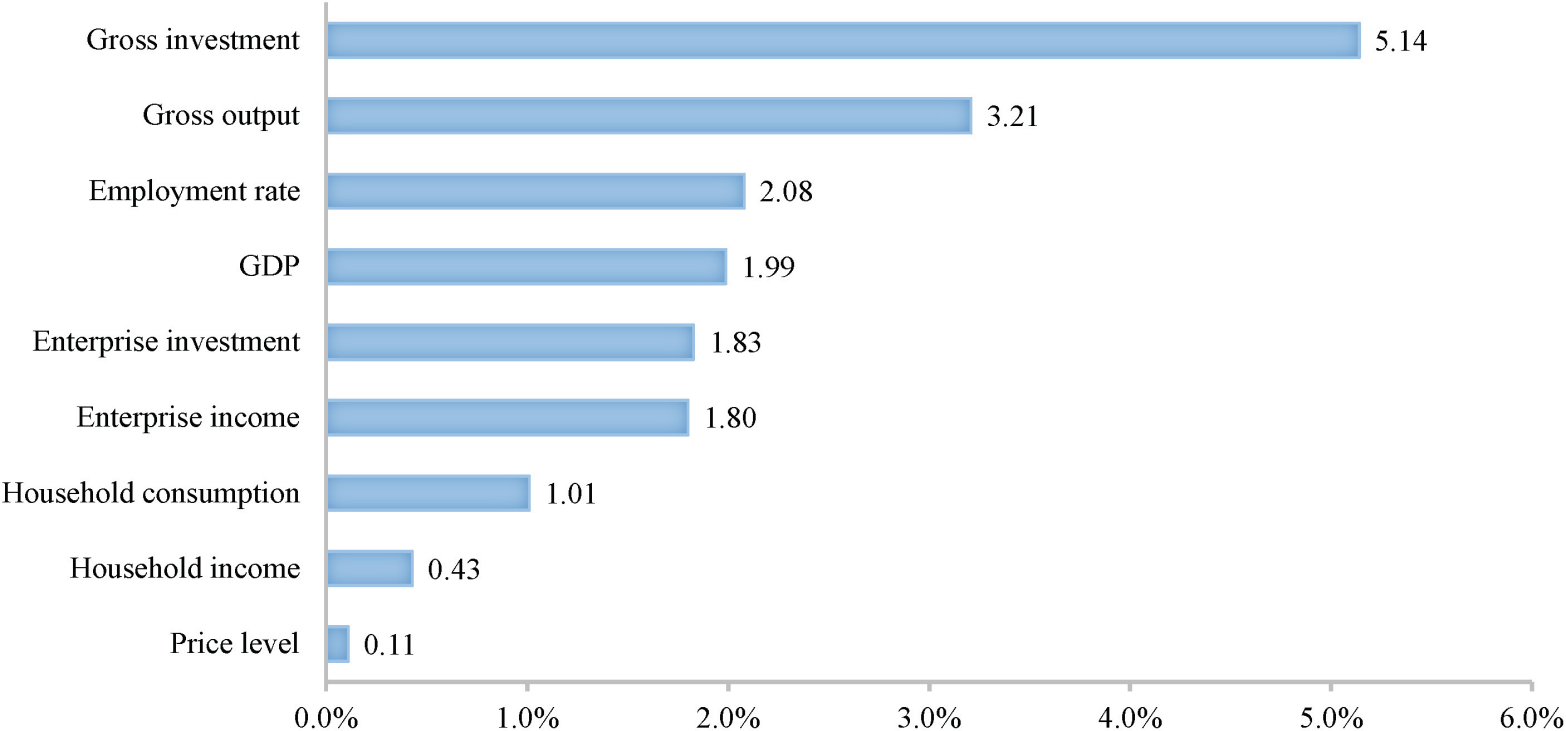
The total effects of monetary policies on macro-economy (Unit: %).

Figures 3, 4 show simulation results of overall effects of fiscal and monetary policies on a macro-economic scale. In general, the two types of policies both exert certain effects in economic recovery. When aiming to improve residents' welfare, fiscal policies are better than monetary policies. Fiscal policies improve household income (+0.95%) and consumption (+2.72%) to an extent two times higher than monetary policies. In addition, expanding government investment in infrastructure construction can also promote formation of infrastructure capital in the long term, thus better meeting people's living needs.

When aiming to promote recovery and development of enterprises, monetary policies are better, improving enterprise investment (+1.83%) while increasing revenue (1.80%). When taking boosting the economy and stabilizing prices as objectives, monetary policies are better than fiscal policies. The former not only increase societal aggregate demand to increase gross investment and consumption to 5.14% and 1.01%, but also improve aggregate societal supply, contributing to a 3.21% increase in gross output. Therefore, monetary policy does not drive inflation. In comparison, fiscal policies boost economic growth mainly by expanding government investment in infrastructure construction. However, infrastructure construction generally takes a long time, so fiscal policies increase aggregate supply significantly less than aggregate demand in a short term.

### Stability test of the CGE model

Stability of the CGE model is the premise to ensuring meaning in its results. In the present research, the stability of the CGE model is tested by changing values of coefficients of the exogenous elasticities. The exogenous elasticities involved here include capital-labor substitution elasticity, substitution elasticity between domestic products and imports, price elasticity of export demands, and expenditure elasticity of residents for different goods. The coefficients of these elasticities are separately increased or decreased by 10% from the pre-set values to check whether the results regarding each policy remain stable.

Table 3 lists the effects of exempting VAT for designated sectors on the macro-economy after changing the coefficients of elasticities of the original model. Limited by the length, only the stability test results of one policy (exempting VAT for designated sectors) are presented here. Stability testing of results of other policies did not change the conclusions of the research. It can be seen from the simulated results that either increasing or decreasing the coefficients exerts slight influences on the original model and the simulation results after adjustment remain similar to the original results, thus verifying the stability of the model.

**Table 3:** Robustness test for exempting VAT for designated sectors (Unit: %).

**Changes relative to the base period**	**Original results**	**The elasticity increases by 10%**	**The elasticity decreases by 10%**
GDP	0.39	0.39	0.38
Gross output	0.17	0.17	0.17
Gross investment	0.01	0.01	0.00
Price level	0.25	0.25	0.25
Employment rate	0.42	0.42	0.42
Household income	0.30	0.31	0.30
Household consumption	0.44	0.44	0.44
Enterprise income	0.41	0.42	0.40
Enterprise investment	0.12	0.12	0.12

### Performance and limitations of the CGE model

The contribution of the study is to add a financial module to the traditional CGE model, which facilitates the comparative analysis of the implementation effects of fiscal and monetary policies. Given the adverse impact of COVID-19 on the social economy, this may be a valuable source of information for the government to take immediate action. However, there are still some limitations in this paper, which need to be improved in the future. In terms of model setting, the article only focuses on the impact of loose fiscal and monetary policies on economic recovery but does not quantitatively analyze the possible impact of COVID-19 on the social economy. It is worth noting that some studies have pointed out that the outbreak of the COVID-19 pandemic may have adverse effects on market expectations and consumption patterns (5, 42). As a result, even though China has adopted loose monetary and fiscal policies, residents and enterprises may still be afraid to consume and invest due to weak expectations. And this will weaken the transmission effects of fiscal and monetary policies in the actual situation. Although the problem is beyond the quantitative scope of the CGE model, it is important to the social economy and needs further attention in the future.

## Conclusions and policy implications

The CGE model is established based on the latest China's Input-Output table and the finance module is added to the standardized CGE model. Compared with the traditional CGE model, this provides a convenient setting for studying effects of loans and lending in the finance market on the overall market equilibrium. Compared with previous research, the research closely conforms to reality. After summarizing fiscal and monetary policies implemented in China during the COVID-19 pandemic in 2020, five representative policies are chosen for simulation analysis, including exempting VAT for designated sectors, expanding government investment, increasing medical care expenditure, cutting loan rates, and increasing the scale of lending. The positive and adverse effects of these policies are elucidated from three perspectives: society, residents, and enterprises. The following conclusions are drawn:

(1) Among various fiscal policies, expanding government investment shows the most significant effect in boosting the economy, which however will also cause price inflation (+0.45%) and crowd out enterprise investment (-0.03%). GDP grows by 0.72% after implementing the policy. The demand side is improved more significantly, with social gross investment and consumption increasing by 4.21% and 1.87% separately. In contrast, the supply side is less significantly improved, and gross output only increases by 0.95%. For residents, expanding government investment directly boosts social aggregate demand, so society has a higher labor demand and household income is improved accordingly (+0.62%).
(2) Among various monetary policies, providing targeted loans to enterprises directly shows a better effect in boosting the economy compared with cutting loan rates across the board. The policy promotes economic recovery from both the supply and demand sides. Gross investment and consumption separately increase by 4.92% and 0.97%, gross output grows by 3.07%, and GDP increases by 1.90%. Different from expanding government investment, increasing the scale of lending has a crowding-in effect on enterprise investment, leading to 1.76% and 1.72% increases in enterprise investment and revenue (it is worth noting that this policy may also incur high costs).
(3) For the choice of fiscal and monetary policies, if taking improving people's welfare as the objective, fiscal policies are better than monetary policies. The former improves household income and consumption to an extent two times higher than the latter, finally enabling 0.95% and 2.72% increases in income and consumption. If taking promoting recovery of enterprises and boosting economy as the objectives, monetary policies perform better, finally increasing enterprise revenue, enterprise investment, and GDP by 1.80%, 1.83%, and 1.99%, respectively.

Based on the above results, the following policy implications are proposed:

(1) For fiscal policies, when expanding government investment, adjustment of the interest rate policy is also necessary to decrease financing costs to enterprises, thus avoiding crowding out of enterprise investment. In addition, it is also necessary to implement policies at an appropriate time. Large-scale government investment is not a permanent solution and government should cease large-scale investment at the right time to avoid overheating the economy, which otherwise may cause inflation.
(2) For monetary policies, cutting loan rates in China does not have significant effects in boosting the macro-economy at present. Therefore, the People's Bank of China firstly needs to promote unimpeded transmission of interest rate policies to promote the market-based reform of interest rates. Secondly, the supervisory mechanism for the flow of special re-lending funds should be strengthened, to guide loans to flow to micro, small, and medium-sized enterprises and enterprises in difficulty that are heavily affected by the pandemic. The support of finance for the real economy should be actively and steadily played, to help enterprises rebuild their capital chain to surmount difficulties arising in the pandemic response.
(3) For formulation of fiscal and monetary policies in the face of major emergencies, it should be oriented by different objectives. Fiscal policies should take improving people's welfare as the objective, while monetary policies should aim to boost the economy and promote recovery of enterprises.

## Data availability statement

The original contributions presented in the study are included in the article/Supplementary material, further inquiries can be directed to the corresponding author/s.

## Author contributions

YW: conceptualization and funding acquisition. YW and YL: methodology and validation. YL: software, data curation, writing and editing, and visualization. Both authors contributed to the article and approved the submitted version.

## Funding

This work was supported by the National Natural Science Foundation of China (Funding No. 71703060) and Humanities and Social Sciences Youth Foundation, Ministry of Education of the People's Republic of China (17YJC790170). The above two funds mainly provide financial assistance in the process of article discussion and language polishing.

## Conflict of interest

The authors declare that the research was conducted in the absence of any commercial or financial relationships that could be construed as a potential conflict of interest.

## Publisher's note

All claims expressed in this article are solely those of the authors and do not necessarily represent those of their affiliated organizations, or those of the publisher, the editors and the reviewers. Any product that may be evaluated in this article, or claim that may be made by its manufacturer, is not guaranteed or endorsed by the publisher.

## Footnotes

^1^Data from the International Monetary Fund, https://www.imf.org/zh/Publications/WEO/Issues/2021/01/26/2021-world-economic-outlook-update.

^2^Data from the Ministry of Finance of the People's Republic of China, http://www.mof.gov.cn/index.htm.

^3^Data from the People's Bank of China, http://www.pbc.gov.cn/goutongjiaoliu/113456/113469/4021012/index.html.

^4^Data pertaining to exempting VAT for designated sectors comes from the Report on the Work of the Government in 2020.

^5^Data pertaining to government investment in traditional infrastructure construction comes from the Central Government's Budget for Expenditure on Capital Construction in 2020, in which central government investment in the transportation sector increases by 20.7% over the previous year.

^6^Data pertaining to government investment in new infrastructure construction comes from the Central Government's Budget for Expenditure on Capital Construction in 2020, in which innovation-driven central government investment in infrastructure has year-on-year growth of 34.5%.

^7^Data pertaining to government medical care expenditure comes from the website of the Ministry of Finance of the People's Republic of China. Therein, funds earmarked for pandemic prevention by financial departments at all levels in 2020 amounted to 116.9 billion yuan. Total government medical care expenditure in 2020 was 1.6797 trillion yuan. Therefore, the medical care expenditure for pandemic prevention was about 1169/16, 797 × 100% ≈ 6.96% of the total.

^8^Data pertaining to the interest rate on corporate loans comes from the Report on China's Monetary Policy Implementation in the Fourth Quarter of 2020. The weighted average interest rate on corporate loans had declined from 5.12% in the same period of 2019 to 4.61% in December 2020.

^9^Data pertaining to the scale of corporate lending comes from the Report on China's Monetary Policy Implementation in the Fourth Quarter of 2020. The scale of lending by enterprises and public institutions had grown by 12.6% as of December 2020 over the previous year.
