# Supplementary material for: How to cushion economic recession caused by the COVID-19 pandemic: Fiscal or monetary policies?
Source: Front Public Health. 2022 Oct 28;10:960655. doi: 10.3389/fpubh.2022.960655 (PMC9650139; doi:10.3389/fpubh.2022.960655)
Supplement: Supplementary file 1 [file Table_2.DOCX]

**Appendix A.** Data base of CGE model in China (unit: 100 million yuan).

| **Row** | **Column** | **Data name** | **Data source** | **Value** |
| --- | --- | --- | --- | --- |
| 1. Activity | 2. Commodity | Total output | Sum of columns for "Activity" items | 225729 |
| 2. Commodity | 1. Activity | Intermediate input | Input-Output table in 2017 | 1438503 |
|  | 5. Resident | Resident consumption | National Bureau of Statistics | 317883 |
|  | 7. Government | Government consumption | Flow-of-funds table in 2017 | 118995 |
|  | 9. Investment | Total investment | Flow-of-funds table in 2017 | 213314 |
|  | 10. Foreign | Export | International balance of payments in 2017 | 164115 |
| 3. Labor | 1. Activity | Labor reward | Flow-of-funds table in 2017 | 423266 |
|  | 10. Foreign | Net labor reward abroad | International balance of payments in 2017 | 1017 |
| 4. Capital | 1. Activity | Capital gain | Flow-of-funds table in 2017 | 303077 |
| 5. Resident | 3. Labor | Labor income | Flow-of-funds table in 2017 | 424283 |
|  | 4. Capital | Capital Income | Calculated by row-column balance | 567 |
|  | 6. Enterprise | Transfer payment | Calculated by row-column balance | 99213 |
|  | 7. Government | Transfer payment | Finance Yearbook of China in 2018 | 31470 |
|  | 8. Bank | Deposit interest income | Flow-of-funds table in 2017 | 94014 |
|  | 10. Foreign | Transfer payment | International balance of payments in 2017 | 1091 |
| 6. Enterprise | 4. Capital | Capital income | Capital gain + Transfer payment from foreign to enterprise | 298842 |
|  | 7. Government | Transfer payment | Finance Yearbook of China in 2018 | 9415 |
|  | 8. Bank | Enterprise loan  Deposit interest income | Flow-of-funds table in 2017 | 143913 |
|  | 10. Foreign | Transfer payment | International balance of payments in 2017 | -3351 |

**Continued Appendix A**

| **Row** | | **Column** | **Data name** | **Data source** | **Value** |
| --- | --- | --- | --- | --- | --- |
| 7. Government | | 1. Activity | Net product tax | Flow-of-funds table in 2017 | 90883 |
|  |  | 2. Commodity | Tariffs | China Statistical Yearbook in 2017 | 3157 |
|  |  | 4. Capital | Investment income factor tax | Calculated by row-column balance | 3668 |
|  |  | 5. Resident | Individual income tax | Flow-of-funds table in 2017 | 58095 |
|  |  | 6. Enterprise | Enterprise income tax | Finance Yearbook of China in 2018 | 32102 |
|  |  | 8. Bank | Deposit interest income | Flow-of-funds table in 2017 | 7752 |
|  |  | 10. Foreign | Transfer payment | International balance of payments in 2017 | -120 |
| 8. Bank | | 5. Resident | Resident savings | Flow-of-funds table in 2017 | 191738 |
|  |  | 6. Enterprise | Enterprise savings  Loan interest payment | Flow-of-funds table in 2017 | 204580 |
|  |  | 7. Government | Government savings | Flow-of-funds table in 2017 | 35684 |
|  |  | 1. Foreign | Foreign savings | Flow-of-funds table in 2017 | -2316 |
| 9. Investment | 6. Enterprise | | Fixed asset investment | Flow-of-funds table in 2017 | 258780 |
|  | 7. Government | | Government investment | Flow-of-funds table in 2017 | 53434 |
|  | 8. Bank | | Bank loan investment | National Bureau of Statistics | 72435 |
| 10. Foreign | 2. Commodity | | Import | Input-Output table in 2017 | 149268 |
|  | 8. Bank | | Deposit interest income | Flow-of-funds table in 2017 | 10715 |

**Note:** All data in the table are nominal values of 2017.

**Source:** *Input-Output table in 2017, China Statistical Yearbook in 2017, Flow-of-funds table in 2017, Finance Yearbook of China in 2018, International balance of payments in 2017*.

**Appendix B**. The balanced macro-SAM table (unit: 100 million yuan).

|  | 1  Activity | 2  Commodity | 3  Labor | 4  Capital | 5  Resident | 6  Enterprise | 7  Bank | 8  Government | 9  Investment | 10  Foreign | 11  Total |
| --- | --- | --- | --- | --- | --- | --- | --- | --- | --- | --- | --- |
| 1 Activity |  | 2256279 |  |  |  |  |  |  |  |  | 2256279 |
| 2 Commodity | 1417518 |  |  |  | 350070 |  |  | 103915 | 384516 | 159847 | 2415866 |
| 3 Labor | 423256 |  |  |  |  |  |  |  |  | 985 | 424242 |
| 4 Capital | 303380 |  |  |  |  |  |  |  |  |  | 303380 |
| 5 Resident |  |  | 424242 | 367 |  | 77085 | 79830 | 26696 |  | 1042 | 609261 |
| 6 Enterprise |  |  |  | 300477 |  |  | 212707 | 11072 |  | -2762 | 521494 |
| 7 Bank |  |  |  |  | 191210 | 156301 |  | 30055 |  | -1820 | 375746 |
| 8 Government | 112126 | 4416 |  | 2536 | 67981 | 28147 | 7183 |  |  | -106 | 222283 |
| 9 Investment |  |  |  |  |  | 259961 | 74010 | 50545 |  |  | 384516 |
| 10 Foreign |  | 155170 |  |  |  |  | 2016 |  |  |  | 157186 |
| 11 Total | 2256279 | 2415866 | 424242 | 303380 | 609261 | 521494 | 375746 | 222283 | 384516 | 157186 |  |

**Note:** The leveling of the macro-SAM table refers to the processing method of Zhao and Wang (40), i.e., using the cross-entropy method to make the row sums equal to the column sums.

**
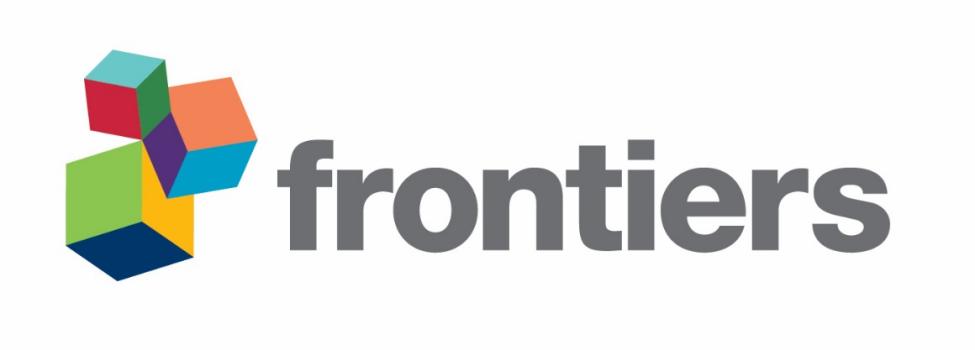
**
